# Supplementary material for: Understanding the Influence of Socioeconomic Variables on Medicinal Plant Knowledge in the Peruvian Andes
Source: Plants (Basel). 2022 Oct 12;11(20):2681. doi: 10.3390/plants11202681 (PMC9608460; doi:10.3390/plants11202681)
Supplement: Supplementary file 1 [file plants-11-02681-s001.zip › plants-1846942-supplementary.pdf]

Supplementary Materials

Table S1. Number of participants interviewed for each socioeconomic variable in 12 localities of the northern Peruvian Andes.

Attributes	Lowlands—Bongará			Lowlands—R. Mendoza			Highlands—Chachapoyas			Highlands—Luya		
	Cuispes	San Carlos	Valera	Huambo	Santa Rosa	Totora	Granada	Olleros	Quinjalca	Longuita	María	Yomblón
**Age**	50	50	50	50	50	50	50	50	50	50	50	50
**Family size**	50	50	50	50	50	50	50	50	50	50	50	50
**Gender**												
Female	25	25	25	25	24	27	25	23	27	26	23	24
Male	25	25	25	25	26	23	25	27	23	24	27	26
**Education**												
No education	3	0	1	0	1	0	14	14	11	10	4	6
Primary education	30	28	34	26	45	43	27	24	18	29	27	25
High education	17	22	15	24	4	7	9	12	11	11	19	19
**Occupation**												
Basic qualification	48	42	43	46	48	49	50	49	49	45	45	44
Medium/high qualification	2	8	7	4	2	1	0	1	1	5	5	6
**Migratory status**												
Non-migrant	28	44	44	13	40	28	44	39	49	30	34	27
Migrant	22	6	6	37	10	22	6	11	1	20	16	23
**Time-in-residence**												
0–15 years	18	17	15	19	18	20	12	10	16	14	18	16
16–30 years	17	12	30	11	14	13	16	10	14	14	11	15
>30 years	15	21	5	20	18	17	22	30	20	22	21	19
**Transport**												
No transport	43	38	33	40	42	35	37	29	35	35	26	32
Animal or bike	4	10	13	9	5	13	10	16	9	12	17	15
Motorbike, car, or similar	3	2	4	1	3	2	3	5	6	3	7	3
**Tools**												
No tools	7	5	17	11	4	3	19	25	27	9	10	11
Basic tools	42	45	30	27	45	43	31	25	22	40	39	36
Semi-automatic tools	1	0	3	12	1	4	0	0	1	1	1	3
**Technological services**												
Radio, mobile phone, TV	19	0	2	3	0	0	23	24	33	19	6	18
Cable TV	22	13	0	23	14	16	19	17	14	17	17	13
Internet	9	37	48	24	36	34	8	9	3	14	27	19
**Farm animals**												
No animals	17	6	20	9	29	7	9	14	20	15	12	11

1–15 animals;	19	23	21	25	12	35	38	30	27	28	21	25
>15 animals	14	21	9	16	9	8	3	6	3	7	17	14
**Farm size**												
0–1 ha	29	22	31	19	20	25	43	50	42	34	24	28
1–5 ha	19	27	13	29	30	23	7	0	7	13	18	18
>5 ha	2	1	6	2	0	2	0	0	1	2	8	4
**House quality**												
Some defects	42	47	2	24	30	46	47	46	50	43	35	40
Good quality, no defects	8	3	48	26	20	4	3	4	0	7	15	10
